# Therapeutic Efficacy and Complication Profile of Monopolar Transurethral Resection of the Prostate (TURP) in the Management of Bladder Outlet Obstruction

**DOI:** 10.7759/cureus.89640

**Published:** 2025-08-08

**Authors:** Muhammad Ashhad Ullah Khalil, Sajid Abbasi, Reena Nawaz, Noor Ul Ain Rashid, Muhammad Anas Ghazi, Yassar Hussain Patujo, Rameez Ahmed Mughal, Faisal Liaquat, Hafiz Ali Raza

**Affiliations:** 1 Urology, Institute of Kidney Diseases, Hayatabad Medical Complex, Peshawar, PAK; 2 Urology, Shaheed Mohtarma Benazir Bhutto Medical University, Larkana, PAK; 3 Trauma and General Surgery, Akhtar Saeed Trust Hospital, Lahore, PAK; 4 Trauma and Orthopedics, North Manchester General Hospital, Manchester, GBR; 5 Urology, Chandka Medical College, Shaheed Mohtarma Benazir Bhutto Medical University, Larkana, PAK; 6 Urology, Benazir Bhutto Hospital, Rawalpindi, PAK; 7 Urology, Sindh Employees Social Security Kidney Centre, Sindh Employees Social Security Institution (SESSI), Karachi, PAK; 8 Agriculture Extension, Muhammad Nawaz Shareef University of Agriculture, Multan, PAK

**Keywords:** benign prostatic hyperplasia, bladder outlet obstruction, functional outcomes, monopolar resection, postoperative complications, turp

## Abstract

Background: Bladder outlet obstruction (BOO) due to benign prostatic hyperplasia (BPH) is a common urological condition in aging men, often requiring surgical intervention for symptom relief.

Objective: To evaluate the therapeutic efficacy and complication profile of monopolar transurethral resection of the prostate (TURP) in patients with BOO.

Methodology: This descriptive observational study was conducted at the Department of Urology, Hayatabad Medical Complex, Peshawar, and Akhtar Saeed Trust Hospital, Lahore, from August 2022 to July 2024. A total of 518 male patients aged ≥ 50 years with BOO secondary to BPH underwent monopolar TURP. Preoperative, intraoperative, and postoperative variables were collected using a standardized proforma. Functional outcomes, including International Prostate Symptom Score (IPSS), maximum urinary flow rate (Qmax), and post-void residual volume (PVR), were assessed at baseline and one, six, and 12 months postoperatively. Complication rates and predictors were also analyzed.

Results: Mean age was 68.42 ± 7.85 years. Significant improvement was observed in IPSS (from 23.74 ± 4.96 to 7.82 ± 2.81), Qmax (from 7.89 ± 2.11 mL/sec to 17.50 ± 4.67 mL/sec), and PVR (from 126.40 ± 34.70 mL to 30.10 ± 16.87 mL) at 12 months (p < 0.001). Early complications included hematuria in 42 patients (8.11%), UTI in 61 (11.78%), and TUR syndrome in six (1.16%). Late complications included urethral stricture (3.67%) and bladder neck contracture (2.12%).

Conclusion: Monopolar TURP is an effective and safe surgical option for managing BOO due to BPH in resource-limited settings.

## Introduction

A common urological disorder that primarily affects older men, bladder outlet obstruction (BOO) is characterized by increased resistance to urine flow through the urethra or bladder neck [1,2]. The most common underlying cause is benign prostatic hyperplasia (BPH), a gradual growth of the prostate gland that causes lower urinary tract symptoms (LUTS) such as urgency, frequency, nocturia, weak stream, and hesitancy, which considerably reduce quality of life [3,4]. Although some individuals may receive symptomatic relief from medication therapy, surgery is still the recommended course of treatment for patients with moderate to severe symptoms or consequences such as renal impairment, bladder stones, or recurrent urine retention [5].

The gold standard surgical therapy for BOO related to BPH has traditionally been considered to be monopolar transurethral resection of the prostate (TURP) [6,7]. In order to relieve the blockage and restore normal urine flow, the treatment entails the endoscopic excision of prostatic tissue utilizing a monopolar electrosurgical loop [8]. Because of its accessibility, affordability, and proven long-term results, monopolar TURP is still often used, particularly in healthcare settings with low resources, even in the face of the development of more recent technologies like bipolar TURP, laser therapy, and less invasive procedures [9].

Monopolar TURP does, however, include the risk of both intraoperative and postoperative problems, much like any invasive operation. These may include urethral stricture, bladder neck contracture, hemorrhage, urinary tract infection, and, in rare instances, transurethral resection syndrome (a potentially serious complication characterized by dilutional hyponatremia, fluid overload, nausea, vomiting, visual disturbances, confusion, or even cardiovascular collapse, resulting from excessive absorption of hypotonic irrigation fluid during the procedure) [10,11]. Optimizing patient outcomes and directing clinical decision-making requires an understanding of the therapeutic effectiveness and safety profile of monopolar TURP in various clinical situations.

The success of monopolar TURP in terms of symptom alleviation, urine flow improvement, and complication rates has to be reevaluated in light of the changing surgical environment and the fact that it is still used in many areas. Using current clinical data, this research adds to the body of literature by providing a thorough assessment of monopolar TURP outcomes in BOO patients.

Research objective

To determine the clinical outcomes and associated complications of monopolar TURP in the management of BOO.

## Materials and methods

Study design and setting

This descriptive observational study was conducted at the Department of Urology, Hayatabad Medical Complex, Peshawar, and Akhtar Saeed Trust Hospital, Lahore, from August 2022 to July 2024. Every eligible patient having monopolar TURP for BOO at this time was evaluated for possible inclusion. To improve dependability and reduce bias, a consistent follow-up strategy and standardized data-collecting procedures were put into place.

Inclusion and exclusion criteria

The research comprised male patients aged 50 years or older who had been clinically diagnosed with BOO as a result of BPH, had undergone monopolar TURP, and gave their informed consent. Patients with BOO from other causes, such as prostate cancer, bladder neck stenosis, or urethral stricture; a history of prior prostate surgery, bipolar TURP, or other surgical procedures; or significant neurogenic bladder or other major urological comorbidities were not included.

Sampling and sample size

A total of 518 patients were enrolled through convenience sampling from eligible inpatient and outpatient populations at the study centers. This approach was selected due to the nature of the study design and the intention to include all consecutive patients meeting the inclusion criteria during the study period. Although no formal sample size calculation was conducted, given the exploratory objectives of this observational study, the final cohort is comparable in size to previous studies assessing TURP outcomes. Hirik et al. [12] evaluated 590 patients (300 monopolar, 290 bipolar TURP), while Bioku et al. [13] reported outcomes in 142 patients undergoing monopolar TURP. These comparisons support the clinical relevance and adequacy of our sample size. Limitations related to sampling and generalizability are acknowledged in the discussion.

Data collection

A standardized and pre-validated proforma was used to gather data prospectively. Age, comorbidities, prostate volume (measured by transrectal ultrasonography), post-void residual volume (PVR), International Prostate Symptom Score (IPSS) [14], and maximal urine flow rate (Qmax) were among the preoperative factors. To reduce surgical variability, all TURP operations were carried out by urologists at the consultant level with a standardized monopolar method. Resection time, expected blood loss, weight of resected prostate tissue, and intraoperative problems were among the intraoperative data that were documented. Duration of catheterization, length of hospital stay, and early complications such as hematuria, urinary tract infection (UTI), TUR syndrome, or clot retention were among the postoperative characteristics evaluated. At one, six, and twelve months after surgery, functional outcomes were reassessed with an emphasis on changes in IPSS, Qmax, and the detection of both immediate and delayed problems, including bladder neck contracture, urethral stricture, and the need for re-intervention. To minimize inter-observer and reporting bias, all clinical evaluations and follow-ups were carried out by qualified research personnel using standardized instruments. At one, six, and twelve months after surgery, functional outcomes were reassessed with an emphasis on changes in IPSS, Qmax, and the detection of both immediate and delayed problems, including bladder neck contracture, urethral stricture, and the need for re-intervention.

Statistical analysis

Data were analyzed using IBM SPSS Statistics for Windows, Version 26 (Released 2018; IBM Corp., Armonk, New York, United States). Descriptive statistics were used to summarize patient demographics, clinical features, surgical findings, and postoperative outcomes. Quantitative variables were expressed as means ± standard deviations (SD), and categorical variables as frequencies and percentages. Preoperative and postoperative outcomes (IPSS, Qmax, and PVR) were compared using paired sample t-tests. Multivariate logistic regression analysis was performed to identify predictors of postoperative complications. A p-value < 0.05 was considered statistically significant.

Ethical approval

The Institutional Review Board (IRB) of Hayatabad Medical Complex, Peshawar, examined and approved the research protocol (639/DOU/HMC/2022). Prior to enrollment, all individuals provided written informed consent. Throughout the research, patient anonymity was rigorously maintained, and all procedures were carried out in compliance with institutional ethical standards and the Declaration of Helsinki.

## Results

Data from 518 male patients with an average age of 68.42 ± 7.85 years were evaluated for the research (Table 1). Around 271 patients (52.32%) had hypertension, 198 patients (38.22%) had diabetes mellitus, and 79 patients (15.25%) had ischemic heart disease. The measured prostate volume was 67.51 ± 18.40 cc on average. The mean PVR was 126.40 ± 34.70 mL, the mean Qmax was 7.89 ± 2.11 mL/sec, and the mean preoperative IPSS was 23.74 ± 4.96.

**Table 1:** Baseline demographic and preoperative clinical characteristics (n = 518)

Category	Parameter	Mean ± SD / Frequency (%)
Demographics	Age (years)	68.42 ± 7.85
	Hypertension	271 (52.32%)
	Diabetes Mellitus	198 (38.22%)
	Ischemic Heart Disease	79 (15.25%)
Prostate Characteristics	Prostate Volume (cc)	67.51 ± 18.40
Urinary Function	International Prostate Symptom Score	23.74 ± 4.96
	Qmax (mL/sec)	7.89 ± 2.11
	Post-void Residual Volume (PVR, mL)	126.40 ± 34.70

Table 2 shows a significant increase in function after TURP. After one month, the mean IPSS dropped from 23.74 to 12.40, to 9.51 at six months, and to 7.82 at twelve months (p < 0.001). After one month, Qmax rose from 7.89 mL/sec to 13.27, 15.91, and 17.50 mL/sec (p < 0.001). At the same follow-up intervals, PVR decreased from 126.40 mL to 62.50, 39.70, and 30.10 mL, respectively (p < 0.001), showing steady improvement in all metrics for all 518 patients.

**Table 2:** Comparison of preoperative and postoperative functional outcomes (n = 518) Comparison of preoperative and postoperative functional outcomes at one, six, and 12 months after TURP (n = 518). Data are presented as mean ± SD. p-values and t-statistics are from paired sample t-tests comparing 12-month values with preoperative baseline. TURP: transurethral resection of the prostate

Outcome Measure	Preoperative	1 Month	6 Months	12 Months	t-value	p-value (12 months)
International Prostate Symptom Score (IPSS)	23.74 ± 4.96	12.40 ± 3.90	9.51 ± 3.22	7.82 ± 2.81	64.1	< 0.001
Qmax (mL/sec)	7.89 ± 2.11	13.27 ± 3.58	15.91 ± 4.02	17.50 ± 4.67	61.3	< 0.001
Post-void Residual Volume (PVR) (mL)	126.40 ± 34.70	62.50 ± 22.44	39.70 ± 18.36	30.10 ± 16.87	66.7	< 0.001

The average operative time was 58.35 ± 13.28 minutes with a mean estimated blood loss of 184.27 ± 43.61 mL and resected prostate weight of 34.18 ± 9.72 grams (Table 3). Postoperatively, the average catheterization duration was 2.76 ± 1.14 days, and the hospital stay was 3.94 ± 1.32 days. Hematuria requiring irrigation occurred in 42 patients (8.11%), UTI in 61 patients (11.78%), clot retention in 25 patients (4.83%), and TUR syndrome in six patients (1.16%).

**Table 3:** Intraoperative and early postoperative outcomes (n = 518)

Parameter	Mean ± SD / Frequency (%)
Operative Time (minutes)	58.35 ± 13.28
Estimated Blood Loss (mL)	184.27 ± 43.61
Resected Prostate Weight (grams)	34.18 ± 9.72
Duration of Catheterization (days)	2.76 ± 1.14
Length of Hospital Stay (days)	3.94 ± 1.32
Hematuria Requiring Irrigation	42 (8.11%)
Urinary Tract Infection (UTI)	61 (11.78%)
Clot Retention	25 (4.83%)
Transurethral Resection (TUR) Syndrome	6 (1.16%)

Among the 518 patients, the most common long-term complication was urethral stricture, followed by bladder neck contracture. A small proportion of patients required re-intervention for any cause, while recurrent LUTS and repeat TURP procedures were infrequent. Statistical analysis confirmed that the incidence of these complications was low overall, underscoring the long-term safety and durability of the procedure (Table 4).

**Table 4:** Late postoperative complications and re-interventions observed during follow-up after TURP (n = 518) p-values and χ² statistics are from chi-square tests comparing observed incidence to expected (null) values.

Complication/Outcome	Frequency (n)	Percentage (%)	Test Statistic (χ²)	p-value
Urethral Stricture	19	3.67	15.2	<0.001
Bladder Neck Contracture	11	2.12	9.8	0.002
Re-intervention (Any Cause)	14	2.70	12.1	<0.001
Recurrent Lower Urinary Tract Symptom (LUTS)	4	0.77	3.6	0.058
Repeat Transurethral Resection of the Prostate (TURP)	3	0.58	2.8	0.094

According to Table 5, statistical analysis, patients over 70 had noticeably greater risks of postoperative complications (OR 1.87; 95% CI: 1.01-3.45; p = 0.044). A significant predictor was diabetes mellitus (OR 2.14; 95% CI: 1.18-3.87; p = 0.011), followed by an operation lasting more than 60 minutes (OR 2.06; p = 0.017), and a blood loss of more than 200 milliliters (OR 2.22; p = 0.009). Although it was not statistically significant, a prostate volume larger than 80 cc demonstrated a favorable tendency (OR 1.63; p = 0.110).

**Table 5:** Multivariate logistic regression analysis (predictors of postoperative complications) (n = 518)

Variable	OR (95% CI)	p-value
Age > 70 years	1.87 (1.01–3.45)	0.044
Diabetes Mellitus	2.14 (1.18–3.87)	0.011
Prostate Volume > 80 cc	1.63 (0.89–2.97)	0.110
Operative Time > 60 min	2.06 (1.14–3.72)	0.017
Estimated Blood Loss > 200 mL	2.22 (1.21–4.10)	0.009

## Discussion

The current research showed that individuals with BOO due to BPH have a substantial improvement in their flow metrics and urine symptoms after monopolar TURP. The mean IPSS improved at 12 months after surgery, from 23.74 ± 4.96 to 7.82 ± 2.81 (p < 0.001). Excellent symptomatic and functional results were also shown by the PVR, which decreased from 126.40 ± 34.70 mL to 30.10 ± 16.87 mL, and the Qmax, which increased from 7.89 ± 2.11 mL/sec to 17.50 ± 4.67 mL/sec. These results are consistent with other research that found that mean IPSS significantly decreased and that Qmax significantly increased at the one-year follow-up after monopolar TURP [8,15].

The average estimated blood loss was 184.27 ± 43.61 mL, the mean operational duration was 58.35 ± 13.28 minutes, and the removed prostate tissue weighed 34.18 ± 9.72 grams. These figures are in line with earlier research that found that tissue resection weights (average 35.6 grams) and operating times (mean 60 minutes) were comparable in patients of monopolar TURP [16]. In line with data from worldwide studies, where the incidence of TUR syndrome with monopolar TURP normally ranges from 0.5% to 2% [17], our cohort's very low intraoperative complication rates, TUR syndrome (1.16%), and clot retention (4.83%) reflect a positive safety profile.

With 42 patients (8.11%) needing irrigation for hematuria and 61 patients (11.78%) suffering from UTI, the early postoperative sequelae were controllable. Although our research found a somewhat higher hematuria rate of 8.11%, these results are similar to those of other studies that revealed UTI rates of 8.06% and hematuria needing irrigation in 4.8% of patients [18]. There were very few long-term side effects: 11 patients (2.12%) had bladder neck contracture, and 19 patients (3.67%) developed urethral stricture. Previous investigations have indicated similar percentages, with urethral strictures occurring in 2.2-9.8% of patients and bladder neck contractures occurring in 0.3-9.2% of cases [10].

Using multivariate analysis, postoperative complications were shown to be independently predicted by diabetes mellitus (OR 2.14, p = 0.011), age > 70 years (OR 1.87, p = 0.044), operational duration > 60 minutes (OR 2.06, p = 0.017), and blood loss > 200 mL (OR 2.22, p = 0.009). These results support earlier research findings that highlighted the influence of surgical complexity and patient comorbidities on TURP outcomes [19].

Study strengths and limitations

This study's comparatively large sample size of 518 patients is one of its main advantages, as it enables a thorough assessment of the functional outcomes and complication rates after monopolar TURP. The findings' internal validity and reliability are improved by the prospective design and use of standardized preoperative and postoperative evaluation instruments, such as IPSS, Qmax, and PVR. Additionally, to reduce operator variability, surgeries were carried out by urologists at the consultant level using a standard surgical protocol. The longitudinal follow-up at one, six, and 12 months adds strength to the study by allowing consistent tracking of symptom improvement and complications over time. Another key strength is the real-world applicability of the findings, as the procedures were performed in resource-limited clinical settings, reflecting outcomes relevant to many urology centers in similar environments.

Direct comparisons are limited by the lack of a comparator group (such as bipolar TURP or medicinal treatment). Although this was a focused single-arm study, we acknowledge that including a comparative arm would have enhanced the interpretability of the outcomes across different TURP modalities. Complications, including sexual dysfunction and long-term outcomes beyond a year, were not examined, which may have yielded a more thorough safety profile. Additionally, potential selection bias due to convenience sampling and the lack of adjustment for non-urological comorbidities (e.g., cardiovascular or neurological conditions) that may affect postoperative recovery are recognized limitations. Lastly, while our findings are relevant to similar practice settings, their generalizability to other populations or healthcare systems with different resources, patient profiles, or surgical protocols should be interpreted with caution.

## Conclusions

Monopolar TURP remains a well-established, effective, and accessible surgical treatment for BPH, particularly in resource-limited settings where newer technologies may not be widely available. Its proven ability to relieve BOO and improve urinary function makes it a valuable option when performed with proper patient selection and adherence to standard surgical protocols. Careful perioperative planning, especially for older patients and those with comorbid conditions such as diabetes, is essential to minimize complications and optimize outcomes. Overall, monopolar TURP continues to offer a favorable balance of efficacy, safety, and cost-effectiveness, reinforcing its role as a cornerstone in the surgical management of LUTS due to prostatic enlargement. Future multicenter studies with larger sample sizes and longer-term follow-up are warranted to further validate these findings and explore comparative effectiveness across surgical modalities.
